# Assessing the Impact of the Pandemic on Treatment Outcomes for Cardiac Arrest Patients Utilizing Mechanical CPR: A Nationwide Population-Based Observational Study in South Korea

**DOI:** 10.3390/jpm14111072

**Published:** 2024-10-24

**Authors:** Jae Hwan Kim, Young Taeck Oh, Chiwon Ahn

**Affiliations:** Department of Emergency Medicine, College of Medicine, Chung-Ang University, Seoul 06974, Republic of Korea; thesult@cauhs.or.kr (J.H.K.); powerfreeze@cauhs.or.kr (Y.T.O.)

**Keywords:** cardiopulmonary resuscitation, cardiac arrest, mechanical device, mechanical CPR, COVID-19

## Abstract

Introduction: Cardiopulmonary resuscitation with mechanical devices (MCPR) was developed to provide high-quality cardiopulmonary resuscitation (CPR) for patients with cardiac arrest. However, the effect of this procedure on treatment outcomes remains controversial. Nevertheless, during the coronavirus disease-19 (COVID-19) pandemic, in-hospital MCPR gained attention, owing to its advantages such as saving medical staff and preventing infection. This study compared the treatment outcomes of in-hospital MCPR and manual CPR for out-of-hospital cardiac arrest (OHCA) patients during the COVID-19 pandemic. Materials and Methods: This retrospective nationwide population-based study was conducted in South Korea. Data were collected from the Out-of-Hospital Cardiac Arrest surveillance database managed by the Korea Disease Control and Prevention Agency. We included adult OHCA patients transported by emergency medical services from 2016 to 2021. The study compared outcomes during the COVID-19 pandemic years (2020–2021) with the preceding non-pandemic years (2018–2019). The primary outcome was survival to hospital discharge, and the secondary outcomes were good neurological outcome and sustained return of spontaneous circulation (ROSC). Results: The entire study included 72,050 patients with OHCA and, in the multivariable analyses, MCPR was associated with lower survival rates compared to manual CPR (AOR 0.63; 95% CI 0.51–0.77; *p* < 0.001). Interestingly, during the COVID-19 pandemic, while MCPR use increased, the survival rate did not differ significantly between the MCPR and manual-CPR groups. Conclusion: Our study findings suggest that while MCPR may offer potential benefits, such as decreased infection risk for healthcare workers, it did not demonstrate superior outcomes compared to manual CPR in our study population.

## 1. Introduction

Sudden cardiac arrests remain an important public health concern [1]. Cardiopulmonary resuscitation (CPR) is a critical intervention for enhancing the survival of patients experiencing cardiac arrest [2]. Therefore, there have been continuous efforts to determine the proper chest-compression depth and rate to provide effective and high-quality CPR [3]. When CPR is performed with human-powered chest compressions (manual CPR), the quality of the chest compressions decreases over time, as the rescuer becomes fatigued [4,5]. In response, various mechanical CPR devices have been developed and used to provide automatic chest compressions [6,7]. However, in previous studies, the notion of whether these devices improve treatment outcomes in patients with cardiac arrest has been controversial [8,9]. Mechanical chest-compression devices provide external chest compressions in place of a human rescuer. In general, mechanical CPR devices can be divided into two categories. based on the mechanism by which they deliver chest compressions [10]. One is to press the thoracic cavity widely by a load-distributing band like AutoPulse^TM^ (Revivant, Sunnyvale, CA, USA), and the other is to point and directly press the sternum by a piston device like LUCAS^TM^ (LUCAS, Redmond, WA, USA) and Thumper^TM^ (Michigan Instruments, Grand Rapids, MI, USA) [10]. Some animal studies have shown that mechanical chest compression can increase coronary perfusion pressure and myocardial blood flow, improving neurologically intact survival [11]. In a manikin study, MCPR resulted in better compliance with CPR guidelines compared to manual CPR. This was especially true during patient transport [12]. As such, while CPR with a mechanical device (MCPR) offers benefits such as supplementing the lacking manpower and ensuring constant chest compressions during CPR, evidence to date has not demonstrated its superiority over manual CPR [10]. Even the most recent CPR guidelines do not routinely recommend MCPR [13,14]. Consequently, MCPR is employed as a supplementary intervention, rather than as an alternative to manual CPR [13,14].

MCPR has disadvantages, such as mechanical trauma caused by the device and increased chest-compression hands-off time, due to device installation [15,16,17]. The pandemic has particularly highlighted the potential advantages of MCPR, including minimizing rescuer exposure to the virus and compensating for manpower shortages during peak periods of medical crisis. In particular, the need for MCPR has been highlighted during disasters such as the Coronavirus disease 2019 (COVID-19) pandemic [18,19]. Despite the risk of rescuers becoming infected with COVID-19 from the transmission generated during CPR, there are no immediate tests for COVID-19 in patients with out-of-hospital cardiac arrest (OHCA). The American Heart Association issued interim guidelines on resuscitation, suggesting that MCPR should be considered to reduce the number of rescuers [20].

This study endeavors to bridge this gap by leveraging recent nationwide data from South Korea to assess the impact of MCPR on the outcomes of patients experiencing OHCA. Particularly, it focuses on the COVID-19 pandemic period, during which the use of prehospital MCPR significantly increased to prevent infections and address understaffing issues, a trend that is likely to have extended to in-hospital MCPR, as well [19,21,22]. By comparing the survival rates to hospital discharge between patients treated with manual CPR and those receiving MCPR, and conducting an analysis to elucidate the pandemic’s effect, this research aims to provide a timely and critical evaluation of MCPR’s utility in contemporary emergency medicine. There have been previous studies that analyzed past data, but in this study, the analysis was carried out with updated information [23].

Our investigation aims to systematically assess the prognostic significance of MCPR during the COVID-19 pandemic, utilizing a comprehensive, nationwide dataset from South Korea. The main goal of our study is to evaluate the impact of MCPR on treatment outcomes for patients experiencing OHCA during the pandemic period. Additionally, we aim to shed light on the evolving emergency care practices during health crises, and offer critical insights into optimal CPR strategies. These insights are intended to support healthcare professionals in making well-informed decisions to improve patient care outcomes now and in the future. Furthermore, this study aims to establish a foundational basis for future guidelines on managing OHCA patients, especially in scenarios involving new infectious-disease outbreaks.

## 2. Materials and Methods

### 2.1. Study Design

This retrospective nationwide population-based observational study evaluated the characteristics of patients with OHCA and the prognostic factors associated with survival-to-hospital-discharge, good neurological outcomes, and the return of spontaneous circulation (ROSC) from January 2016 to December 2021, using the Out-of-Hospital Cardiac Arrest Surveillance (OHCAS) database (managed by the Korea Disease Control and Prevention Agency (KDCA)). The database includes all patients with acute OHCA transferred to medical institutions via the EMS (approximately 30,000 patients per year).

In South Korea, public EMSs are managed by the government (National Fire Agency, comprising 19 fire station headquarters), and are provided 24 h a day, 365 days a year [24]. Before visiting the hospital, the paramedics used an automated external defibrillator (AED) to perform CPR. CPR can be stopped, or advanced airway methods can be used, under the supervision of a doctor. However, advanced cardiac life-support drugs cannot be used. After the arrival of victims, each hospital has a policy for administering resuscitation treatments during hospitalization and after ROSC. The EMS data register and hospital medical records were used to obtain patient information from the OHCAS database. Medical-record investigators from the KCDA visited the medical facilities to review the patients’ medical records and to verify several items, in accordance with the Utstein-style [25] and Resuscitation Outcomes Consortium Project [26].

### 2.2. Participants

This study included all adult patients with OHCA transported via the EMSs who were older than 18 years between January 2016 and December 2021. The intervention group comprised patients who underwent MCPR in the emergency department using various devices. The control group included patients who underwent manual CPR. We excluded patients younger than 18 years-old, cases with non-medical causes such as drowning, trauma, poisoning, or hanging, and cases in which CPR was not performed in the emergency department (patients with ROSC before arrival at the medical facility, death on arrival, patients with “do not resuscitate” orders, etc.). Of the total data, 2020 and 2021 were set as the COVID-19 pandemic period and the two previous years, 2018 and 2019, were set as the pre-pandemic period.

### 2.3. Outcome Measures

The primary outcome was survival-to-hospital-discharge, defined as normal discharge or transfer to another medical facility for long-term care after acute treatment. Secondary outcomes were good neurological outcomes and return of spontaneous circulation. Neurological outcomes were categorized using the Cerebral Performance Category (CPC) score, and good neurological outcomes were defined as CPC scores of 1 and 2.

### 2.4. Variables

The primary concern was the use of a mechanical resuscitation device during CPR in the emergency room for patients with OHCA. According to the Utstein style, several variables were collected, including age, sex, place of arrest (public and non-public), whether the arrest was witnessed, whether bystander CPR was performed, initial cardiac rhythm at scene (non-shockable vs. shockable), cause of arrest(cardiac origin; cause of cardiac arrest was due to failure of the heart itself vs. non-cardiac origin), whether defibrillation was performed, and whether advanced interventions were performed, such as percutaneous coronary intervention (PCI), target temperature management (TTM), pacemaker, and extracorporeal membrane oxygenation (ECMO).

### 2.5. Subgroup Analysis

Subgroup analyses were conducted according to the mechanical CPR devices (AutoPulse^TM^, LUCAS^TM^ and Thumper^TM^). This subgroup analysis utilized data from the entire study period (2016–2021) to ensure a sufficient sample size. The AutoPulse^TM^ compresses a wider area of chest by the load-distributing band. The Thumper^TM^ compresses the chest by a piston actuated by pneumatic pressure. These two devices are not capable of decompressing the chest. LUCAS^TM^ applies pointed compression to the chest by a cup-shaped piston, which adheres to the patient’s chest surface to induce active decompression [10].

### 2.6. Statistical Analyses

Categorical variables were analyzed using Pearson’s chi-square and Fisher’s exact tests. Continuous variables were analyzed using an independent samples *t*-test for parametric data and the Mann–Whitney U test for non-parametric data. The Shapiro–Wilk test was used to assess data normality. Multivariable analysis using logistic regression with backward elimination was additionally performed using all statistically significant covariates from the univariate analysis. Following the stepwise elimination of factors in the regression, only the factors that optimized the model’s coefficient of determination remained. Additionally, we performed the propensity score matching (PSM) for the pandemic population between the manual-CPR and mechanical-CPR groups. Propensity scores were computed to 10 decimal places. The closest non-exposure group members in each model were matched to patients in the mechanical-CPR group with a propensity score-difference threshold of less than 1 × 10^−9^. No recurrence was observed in the manual-CPR group. For all data, a *p*-value of less than 0.05 was considered statistically significant. Data were analyzed using R (version 4.3.0; the R Foundation for Statistical Computing, Vienna, Austria).

### 2.7. Ethics Statement

The study protocol was approved by the Institutional Review Board of the Chung-Ang University Hospital, in May 2023 (IRB No. 2305-006-19469). The requirement for informed consent was waived because of the retrospective nature of the study and the use of anonymous clinical data. The Korea Disease Control and Prevention Agency approved the use of the data for this study.

## 3. Results

### 3.1. Patients’ Characteristics

**Figure 1:**
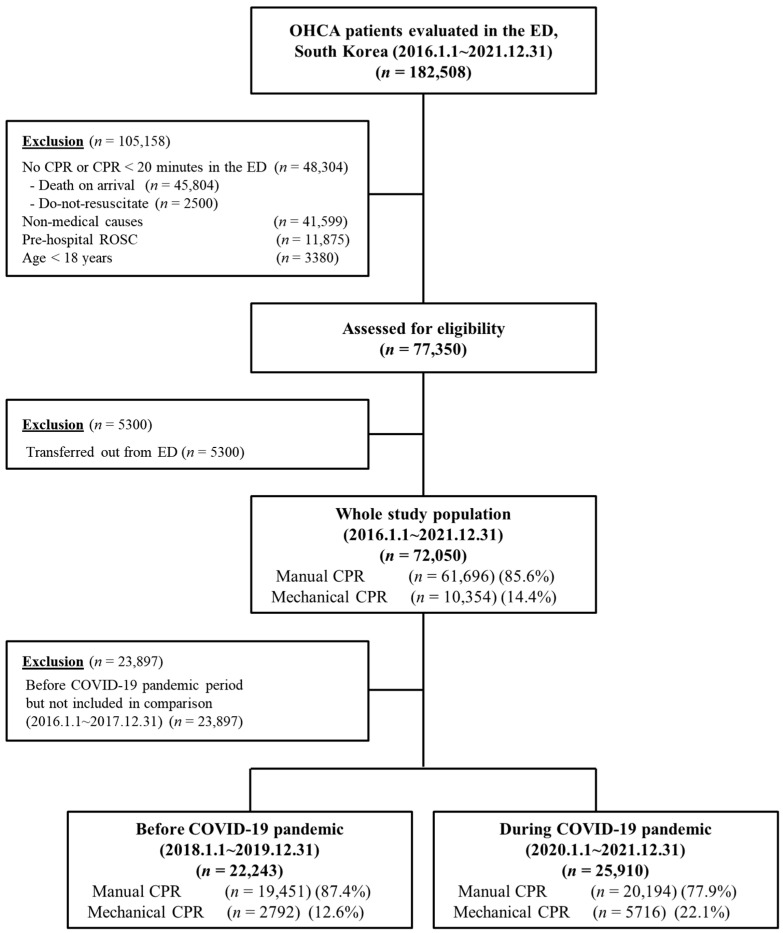
Comparison of patient characteristics in the manual-CPR and mechanical-CPR groups.

We identified 182,508 patients who had experienced OHCAs and assessed 77,350 patients for eligibility after excluding 105,158 patients with traumatic and unknown causes, CPR less than 20 min, do-not-resuscitate orders, ROSC before arrival at the hospital, and age < 18 years. Finally, 72,050 patients were included in our analysis, after excluding patients who were transferred. The patients were divided into two groups, based on the number of patients in each receiving manual or mechanical CPR. A total of 61,696 patients received manual CPR and 10,354 patients received mechanical CPR (Figure 1). Their mean age was 69.4 ± 14.8 years, and 56.5% of patients were male. There were some significant different prehospital factors between manual CPR and MCPR, such as bystander CPR (76.3% vs. 69.5%; *p* < 0.001), cardiac origin arrest (94.2% vs. 93.4%; *p* = 0.003), shockable EKG rhythm (14.9% vs. 13.3%; *p* < 0.001) and prehospital defibrillation (21.7% vs. 19.8%; *p* < 0.001), respectively. In-hospital factors between manual CPR and MCPR, such as TTM (3.5% vs. 4.8%; *p* < 0.001) and ECMO (1.5% vs. 2.9%; *p* < 0.001, respectively,) showed significant differences (Table 1).

### 3.2. Outcomes: Whole Study Period

**Figure 2:**
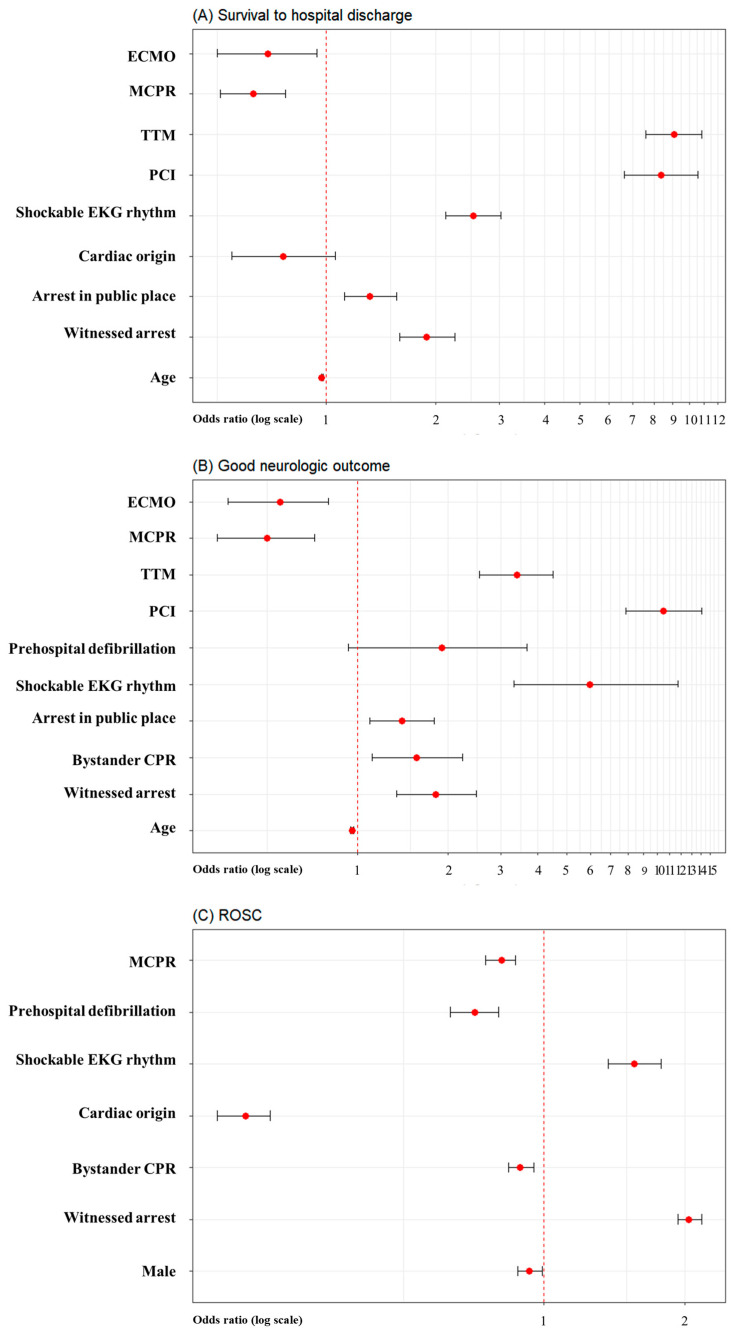
Forest plot depicting multivariable logistic regression analysis for mechanical CPR. (**A**) Survival-to-hospital-discharge. (**B**) Good neurologic outcome. (**C**) ROSC.

Survival-to-hospital-discharge (4.5% vs. 3.7%; *p* < 0.001), good neurological outcomes (1.7% vs. 1.1%; *p* < 0.001), and ROSC (42.9% vs. 40.8%; *p* < 0.001) rates were significantly lower in the MCPR group than in the manual-CPR group (Table 1). Multivariable logistic regression analysis revealed that age, witnessed arrest, arrest in a public place, shockable EKG rhythm, percutaneous coronary intervention, TTM, MCPR, and ECMO were independent risk factors for survival-to-hospital-discharge (Supplementary Table S1) and good neurological outcomes (Supplementary Table S2). Male, witnessed arrest, bystander CPR, cardiac origin, shockable EKG rhythm, prehospital defibrillation, and MCPR were independently associated with ROSC (Supplementary Table S3). MCPR was significantly associated with lower survival-to-hospital-discharge (adjusted odds ratio (AOR) 0.63; 95% confidence interval (CI) 0.51–0.77; *p* <.001) (Figure 2A). Also, MCPR was significantly associated with poor neurologic outcome and ROSC (AOR 0.50; 95% CI 0.34–0.72; *p* < 0.001 and AOR 0.81, 95% CI 0.75–0.87; *p* < 0.001, respectively) (Figure 2B,C).

### 3.3. Outcomes: The COVID-19 Pandemic vs. Before the Pandemic

**Figure 3:**
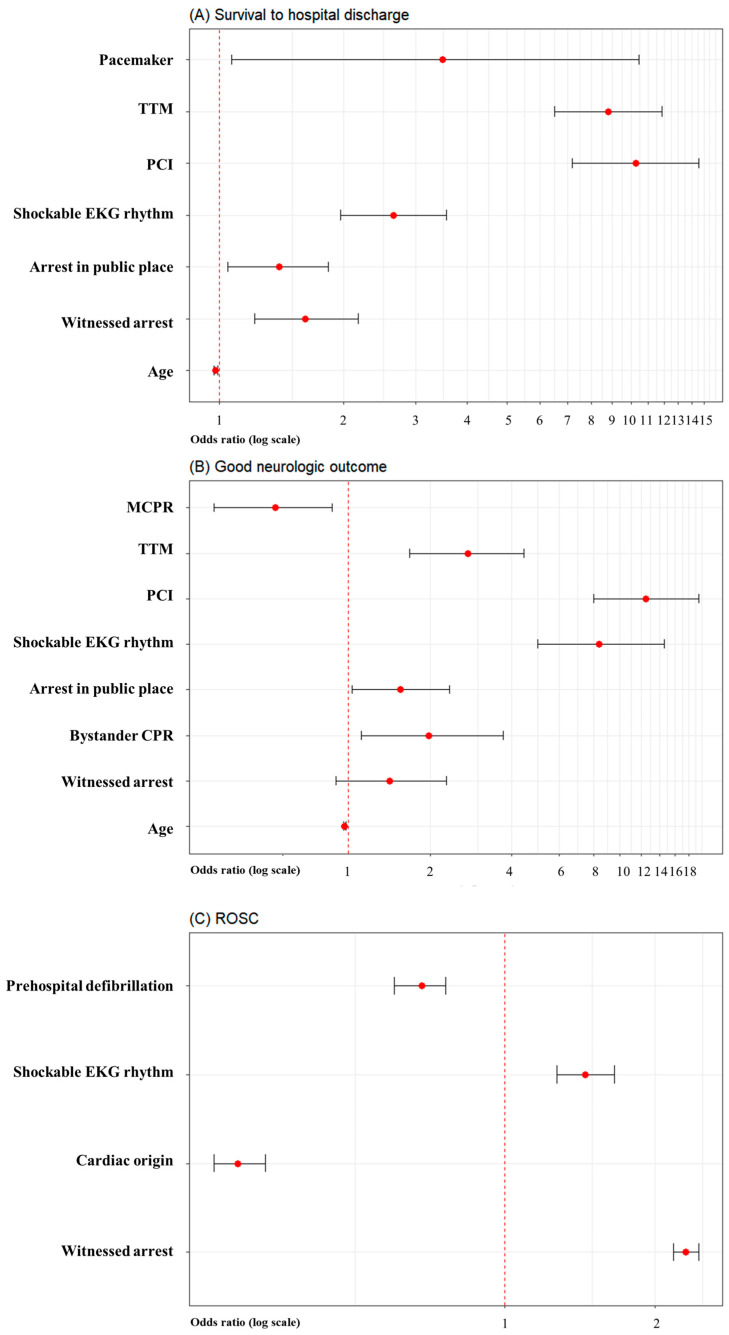
Forest plot depicting COVID-19 pandemic analysis of multivariate logistic regression analysis for mechanical CPR. (**A**) Survival-to-hospital-discharge. (**B**) Good neurologic outcome. (**C**) ROSC.

In an analysis comparing the pre-pandemic and pandemic periods, the frequency of MCPR was 12.6% (2792/22,243) before the pandemic and 22.1% (5716/25,910) during the pandemic (*p* < 0.001). In the pre-COVID-19-pandemic group, survival-to-hospital-discharge, good neurologic outcomes, and ROSC were all significantly reduced with MCPR, compared to manual CPR. In the COVID-19-pandemic group, good neurological outcomes were significantly reduced with MCPR, but ROSC and survival-to-hospital-discharge were not significantly different between the MCPR and manual-CPR groups (Table 2). In the COVID-19 group, MCPR was not significantly associated with survival-to-hospital-discharge (Figure 3A). However, MCPR was significantly associated with poor neurologic outcome (AOR 0.54; 95% CI 0.32–0.87; *p* = 0.014) (Figure 3B) in the COVID-19 pandemic. MCPR was not significantly associated with ROSC (Figure 3C).

The PSM cohort extracted during the pandemic manual-CPR group was larger than the MCPR group. There were 5716 patients in the matching group. Both groups showed a well-balanced distribution of factors, except for PCI. In the PSM cohort, ROSC, survival, and neurological outcomes were significantly reduced with MCPR (Table 3).

### 3.4. Subgroup Analysis: Types of Mechanical CPR Devices

Across all three mechanical CPR devices, the use of mechanical CPR devices did not appear to significantly affect survival-to-hospital-discharge, compared with manual CPR (4.0% vs. 3.3%; *p* = 0.598, 4.0% vs. 3.5%; *p* = 0.153, and 4.0% vs. 7.7%; *p* = 0.053) (Supplementary Tables S4–S6).

## 4. Discussion

The use of mechanical CPR devices is a subject of ongoing debate in the medical community, particularly regarding their effectiveness and impact on treatment outcomes. This study aimed to investigate the impact of in-hospital MCPR on the treatment outcomes of OHCA patients, comparing it with manual CPR, using nationwide population-based data from South Korea. During the COVID-19 pandemic, interim guidelines for the treatment of patients with OHCA were distributed by various organizations, and MCPR was recommended owing to staff shortages and to prevent infection. As a result, MCPR was used more frequently, and we hypothesized that the impact of MCPR on the prognosis of OHCA patients would differ. We compared the prognosis of MCPR with that of manual CPR before and after the COVID-19 pandemic. This was done to provide a basis for future guidelines for the treatment of OHCA patients in the event of a new infectious-disease outbreak.

An analysis of CPR data from OCHA in South Korea between 2016 and 2021 revealed that survival, neurological outcomes, and ROSC were poorer in patients who received MCPR during the hospital stage compared to those who received manual CPR. In multivariate analysis adjusting for various variables, age, witnessed status, occurrence of cardiac arrest in public places, and shockable rhythm were the major variables affecting survival rate. MCPR was significantly associated with a lower survival rate. In the analysis before the COVID-19 pandemic, the survival rate, neurological outcome, and ROSC all showed negative outcomes with MCPR, compared to manual CPR. During the COVID-19 pandemic, the survival rate did not differ significantly between the two groups. Although PSM analysis showed that the MCPR group had lower survival-to-hospital-discharge, in the multivariable logistic analysis, MCPR was not an influencing factor for survival-to-hospital-discharge during the COVID-19 pandemic. It is necessary to note that the impact of the MCPR during the pandemic showed differences from that prior to the pandemic. Based on the results of this study, it cannot be concluded whether or not the application of MCPR improved treatment outcomes.

Before the pandemic, the survival rate of MCPR was 3.8%, and during the pandemic, it was approximately 3.6%. However, with manual CPR, the survival rate was 4.9% before the pandemic and it decreased to approximately 4.0% during the pandemic. This indicates that manual CPR during the pandemic differed from that performed previously. During the COVID-19 pandemic, healthcare workers had been advised to wear personal protective equipment during in-hospital CPR for safety and to prevent the spread of the virus, and to perform resuscitations with fewer staff [27]. This placed an additional burden on the resuscitation efforts, which may have had a negative impact on factors affecting patient outcomes. This could explain why manual CPR did not show a significant difference in survival rate compared to MCPR during the pandemic period.

Additionally, before the pandemic, the MCPR rate was 12.6%, increasing significantly to 22.1% during the pandemic. Before the pandemic, manual CPR was primarily performed, and MCPR was used as an alternative when CPR was prolonged or rescuer fatigue was evident. In contrast, during the pandemic, MCPR was actively applied from the beginning, due to the overall shortage of medical resources and the need to protect medical staff from infection [19], and interim guidelines recommending its application had led to more widespread use of this technique [20,28,29]. As a characteristic of the pandemic, Lim et al. in Korea showed an increase in the use of MCPR in prehospital settings [21], which was the first study to confirm that MCPR was applied at a high frequency in patients admitted to the hospital with OHCA.

Although the findings from our study show reduced survival and neurological outcomes with MCPR, it is important to compare these results with findings from other studies. A meta-analysis of previous randomized controlled trials comparing MCPR with manual CPR found no statistically significant differences between MCPR and manual CPR in treatment outcomes, including ROSC, survival-to-hospital-discharge, and good neurological outcomes [30]. El-Menya et al. conducted an umbrella review of systematic reviews comparing MCPR and manual CPR, and concluded that they could not provide sufficient evidence that MCPR is superior to manual CPR [8] Other studies have indicated that MCPR might be useful in specific scenarios, such as prolonged resuscitation or during transport. However, our findings do not support these advantages in the context of the COVID-19 pandemic, where MCPR did not show a survival benefit.

Kim et al. found that, until 2016, AutoPulse^TM^ was the most common mechanical CPR device used in South Korean hospitals [23]; however, more recently, LUCAS^TM^ has been widely used (Supplementary Figure S1). However, when comparing each device with manual CPR in terms of survival, there was no significant difference, showing the same trend as that in previous studies. LUCAS^TM^ differs from other devices, in that it can be performed according to CPR guidelines in terms of compression rate and depth [31]. The devices are also relatively simple to apply, and have a relative advantage in terms of side effects, which has made them preferred in recent years [32].

Previous studies have shown that in-hospital cardiac arrest (IHCA) cases tend to have specific characteristics and result in better clinical outcomes compared to OHCA cases [33,34]. It is important to note that our study only included patients who experienced OHCA and received CPR before hospital admission. Pure IHCA cases were not included in this analysis, due to the limitations of the OHCAS database. This database focuses exclusively on OHCA patients, and thus we could not investigate subgroup analyses between in-hospital and out-of-hospital CPR patients. As in-hospital cardiac arrest (IHCA) cases may benefit from immediate CPR and a systematic CPR team, the outcomes could differ significantly compared to OHCA cases. We acknowledge this as a limitation of the study, and future research, including both IHCA and OHCA data, is necessary to fully evaluate the effectiveness of mechanical CPR devices across different settings. This study has some limitations. First, the medical environment differs in each country and region, and it is difficult to generalize the results to all situations, as this study was conducted in South Korea. Especially in the analysis of the pandemic, South Korea has a relatively well-controlled COVID-19 situation; therefore, the results have been different in places with different patterns of COVID-19 prevalence. Second, this is a retrospective study, using the OHCAS database. We were unable to measure or adjust for certain factors that could potentially affect OHCA outcomes, such as EMS response times, socioeconomic status, patient history, and the quality of in-hospital CPR, due to the lack of these data in the database. Additionally, important clinical factors that could influence patient outcomes were not available in our database. Laboratory and clinical data could have provided further insights into the impact of these conditions on survival and neurological outcomes in cardiac arrest patients. The absence of such detailed clinical data limits the scope of our analysis, and future studies that incorporate these parameters are needed to better understand their effects on CPR outcomes. Third, the COVID-19 pandemic period included in the sub-analysis was limited to 2020–2021, which is the early- to mid-pandemic period, and is therefore not representative of the entire pandemic. In particular, South Korea experienced an increase in mortality among COVID-19 patients in the first half of 2022 during the early stages of the omicron variant, which was not reflected. This needs to be further analyzed once the raw data are released.

## 5. Conclusions

In conclusion, it is clear that MCPR did not demonstrate superior outcomes to manual CPR in our study population. Despite the potential benefits of MCPR during the COVID-19 pandemic, such as reduced infection risk for healthcare professionals, these might not outweigh the importance of patient outcomes. Our study highlights the importance of thoughtful decision-making when selecting MCPR or manual CPR. It is crucial to note that our findings do not definitively favor one method over the other in terms of patient survival and neurological outcomes. Further research is needed to explore safe and effective CPR methods that can also protect healthcare workers without compromising patient care.

## Figures and Tables

**Table 1:** Comparison of patient characteristics for manual and mechanical CPR.

	Manual CPR (*n* = 61,696)	Mechanical CPR (*n* = 10,354)	Total (*n* = 72,050)	*p* Value
Male	39,617 (64.2%)	6775 (65.4%)	46,392 (64.4%)	0.017
Age, years	69.3 ± 14.8	69.5 ± 14.8	69.4 ± 14.8	0.277
Witnessed arrest	34,619 (58.3%)	5844 (58.1%)	40,463 (58.3%)	0.67
Bystander CPR	14,761 (23.9%)	3612 (34.9%)	18,373 (25.5%)	<0.001
Arrest in public place	9200 (18.5%)	1560 (18.1%)	10,760 (18.4%)	0.47
Cardiac origin	58,089 (94.2%)	9672 (93.4%)	67,761 (94.0%)	0.003
Shockable EKG rhythm	9138 (14.9%)	1370 (13.3%)	10,508 (14.7%)	<0.001
Prehospital defibrillation	13,352 (21.7%)	2044 (19.8%)	15,396 (21.4%)	<0.001
PCI	1571 (2.5%)	277 (2.7%)	1848 (2.6%)	0.46
TTM	2175 (3.5%)	494 (4.8%)	2669 (3.7%)	<0.001
Pacemaker	194 (0.3%)	34 (0.3%)	228 (0.3%)	0.89
ECMO	951 (1.5%)	303 (2.9%)	1254 (1.7%)	<0.001
ROSC	26,443 (42.9%)	4220 (40.8%)	30,663 (42.6%)	<0.001
Survival-to-hospital-discharge	2804 (4.5%)	382 (3.7%)	3186 (4.4%)	<0.001
Good neurologic outcome	1041 (1.7%)	113 (1.1%)	1154 (1.6%)	<0.001

Values are presented as the means ± standard deviations and frequency (proportion). CPR, cardiopulmonary resuscitation; PCI, percutaneous coronary intervention; TTM, targeted temperature management; ECMO, extracorporeal membrane oxygenation; ROSC, return of spontaneous circulation.

**Table 2:** Comparison between manual CPR and mechanical CPR during the COVID-19 pandemic with before the pandemic.

Period	During COVID-19 Pandemic			Before COVID-19 Pandemic		
Chest-Compression Method	Manual CPR (*n* = 20,194)	Mechanical CPR (*n* = 5716)	*p* Value	Manual CPR (*n* = 19,451)	Mechanical CPR (*n* = 2792)	*p* Value
Male	12,883 (63.8%)	3719 (65.1%)	0.081	12,493 (64.2%)	1815 (65.0%)	0.434
Age, years	70.2 ± 14.7	70.1 ± 14.7	0.540	69.6 ± 14.9	69.3 ± 15.1	0.74
Witnessed arrest	11,615 (59.2%)	3302 (59.3%)	0.938	10,577 (56.6%)	1484 (55.0%)	0.125
Bystander CPR	5245 (26.0%)	2100 (36.7%)	<0.001	4810 (24.7%)	899 (32.2%)	<0.001
Arrest at public place	2718 (17.0%)	770 (16.4%)	0.351	2936 (19.5%)	459 (19.9%)	0.651
Cardiac origin	19,049 (94.3%)	5312 (92.9%)	<0.001	18,205 (93.6%)	2609 (93.4%)	0.796
Shockable EKG rhythm	2658 (13.3%)	703 (12.4%)	0.093	2907 (15.0%)	393 (14.1%)	0.218
Prehospital defibrillation	3937 (19.5%)	1064 (18.6%)	0.141	4174 (21.5%)	580 (20.8%)	0.410
PCI	505 (2.5%)	160 (2.8%)	0.225	560 (2.9%)	69 (2.5%)	0.248
TTM	722 (3.6%)	297 (5.2%)	<0.001	760 (3.9%)	114 (4.1%)	0.693
Pacemaker	54 (0.3%)	18 (0.3%)	0.646	54 (0.3%)	8 (0.3%)	1.000
ECMO	359 (1.8%)	156 (2.7%)	<0.001	319 (1.6%)	93 (3.3%)	<0.001
ROSC	8612 (42.6%)	2405 (42.1%)	0.449	8611 (44.3%)	1089 (39.0%)	<0.001
Survival-to-hospital- discharge	808 (4.0%)	210 (3.7%)	0.278	937 (4.8%)	101 (3.6%)	0.006
Good neurologic outcome	317 (1.6%)	66 (1.2%)	0.025	345 (1.8%)	26 (0.9%)	0.002

Values are presented as the means ± standard deviations and frequency (proportion). CPR, cardiopulmonary resuscitation; PCI, percutaneous coronary intervention; TTM, targeted temperature management; ECMO, extracorporeal membrane oxygenation; ROSC, return of spontaneous circulation.

**Table 3:** Comparison of patient characteristics and treatment outcomes in manual and mechanical CPR during the COVID-19 pandemic after the propensity score matching.

Chest-Compression Method	Manual CPR (*n* = 5716)	Mechanical CPR (*n* = 5716)	*p* Value
Male	3701 (64.7%)	3719 (65.1%)	0.739
Age, years	70.0 ± 14.5	70.1 ± 14.7	0.892
Witnessed arrest	3320 (57.8%)	3302 (59.3%)	0.747
Bystander CPR	2129 (37.2%)	2100 (36.7%)	0.588
Arrest at public place	778 (13.6%)	770 (13.5%)	0.848
Cardiac origin	5306 (92.8%)	5312 (92.9%)	0.856
Shockable EKG rhythm	707 (12.4%)	703 (12.3%)	0.932
Prehospital defibrillation	1030 (18.0%)	1064 (18.6%)	0.425
PCI	122 (2.1%)	160 (2.8%)	0.026
TTM	291 (5.1%)	297 (5.2%)	0.832
Pacemaker	10 (0.2%)	18 (0.3%)	0.185
ECMO	126 (2.2%)	156 (2.7%)	0.080
ROSC	2513 (44.0%)	2405 (42.1%)	0.043
Survival-to-hospital- discharge	267 (4.7%)	210 (3.7%)	0.009
Good neurologic outcome	106 (1.9%)	66 (1.2%)	0.003

Values are presented as the means ± standard deviations and frequency (proportion). CPR, cardiopulmonary resuscitation; PCI, percutaneous coronary intervention; TTM, targeted temperature management; ECMO, extracorporeal membrane oxygenation; ROSC, return of spontaneous circulation.

## Data Availability

The datasets generated during the current study are available from the corresponding author on reasonable request.

## Supplementary Materials

The following supporting information can be downloaded at: https://www.mdpi.com/article/10.3390/jpm14111072/s1.
