# Supplementary material for: Assessing the Impact of the Pandemic on Treatment Outcomes for Cardiac Arrest Patients Utilizing Mechanical CPR: A Nationwide Population-Based Observational Study in South Korea
Source: J Pers Med. 2024 Oct 24;14(11):1072. doi: 10.3390/jpm14111072 (PMC11595693; doi:10.3390/jpm14111072)
Supplement: Supplementary file 1 [file jpm-14-01072-s001.zip › Supplementary material.pdf]

LEGENDS FOR Supplementary material

Supplementary Table S1. Univariate and multivariable logistic regression analyses of survival to hospital discharge.

Supplementary Table S2. Univariate and multivariable logistic regression analyses of good neurologic outcomes.

Supplementary Table S3. Univariate and multivariable logistic regression analyses of ROSC.

Supplementary Table S4. Comparison between AutoPulse^{TM} and manual CPR.

Supplementary Table S5. Comparison between LUCAS^{TM} and manual CPR.

Supplementary Table S6. Comparison between Thumper^{TM} and manual CPR.

Supplementary Table S7. Univariate and multivariable logistic regression analyses of survival to hospital discharge during the COVID-19 pandemic.

Supplementary Table S8. Univariate and multivariable logistic regression analyses of good neurological outcomes during the COVID-19 pandemic.

Supplementary Table S9. Univariate and multivariable logistic regression analyses of ROSC during COVID-19 pandemic.

Supplementary Figure S1. Number of in-hospital MCPR uses for OHCA patients by chest compression device

Supplementary Table S1. Univariate and multivariable logistic regression analyses of survival to hospital discharge.

Factor	Univariate OR (95% CI)	*P* value	Adjusted OR (95% CI)	*P* value
Male	1.45 (1.34–1.57)	< .001	–	–
Age, years	0.97 (0.97–0.97)	< .001	0.97 (0.97–0.98)*	< .001
Witnessed arrest	3.35 (3.06–3.68)	< .001	1.89 (1.59–2.26)*	< .001
Bystander CPR	1.19 (1.03–1.37)	.02	–	–
Arrest in public place	1.57 (1.43–1.71)	< .001	1.32 (1.12–1.56)*	< .001
Cardiac origin	0.91 (0.79–1.05)	0.18	0.76 (0.55–10.06)*	.09
Shockable EKG rhythm	4.58 (4.25–4.93)	< .001	2.54 (2.13–30.02)*	< .001
Prehospital defibrillation	3.05 (2.84–3.28)	< .001	–	–
PCI	19.41 (17.54–21.47)	< .001	8.36 (6.61–10.56)*	< .001
TTM	15.78 (14.42–17.26)	< .001	90.07 (7.59–10.81)*	< .001
Pacemaker	8.03 (5.93–10.72)	< .001	–	–
MCPR	0.80 (0.72–0.90)	< .001	0.63 (0.51–0.77)*	< .001
ECMO	6.23 (5.40–7.15)	< .001	0.69 (0.50–0.94)*	.02

Adjusted for sex, age, witnessed arrest, bystander CPR, place of arrest, cause of arrest, shockable EKG rhythm, prehospital defibrillation, PCI, TTM, Pacemaker, MCPR and ECMO. * Factors included in the final logistic regression model for survival to discharge. OR, odds ratio; CI, confidence interval; CPR, cardiopulmonary resuscitation; PCI, Percutaneous coronary intervention; TTM, Targeted temperature management; MCPR, Mechanical cardiopulmonary resuscitation; ECMO, extracorporeal membrane oxygenation.

Supplementary Table S2. Univariate and multivariable logistic regression analysis of good neurologic outcome

Factor	Univariate OR (95% CI)	*P* value	Adjusted OR (95% CI)	*P* value
Male	2.11 (1.83–2.43)	< .001	–	–
Age, years	0.95 (0.95–0.95)	< .001	0.96 (0.95–0.97)*	< .001
Witnessed arrest	4.66 (3.94–5.55)	< .001	1.82 (1.35–2.49)*	< .001
Bystander CPR	1.98 (1.53–2.61)	< .001	1.57 (1.12–2.24)*	.011
Arrest in public place	20.00 (1.74–2.30)	< .001	1.41 (1.10–1.80)*	.007
Cardiac origin	2.48 (1.75–3.67)	< .001	–	–
Shockable EKG rhythm	13.41 (11.82–15.25)	< .001	5.96 (3.33–11.69)*	< .001
Prehospital defibrillation	7.94 (70.02–90.00)	< .001	1.91 (0.93–3.67)*	.06
PCI	34.97 (30.73–39.77)	< .001	10.50 (7.85–140.04)*	< .001
TTM	9.67 (8.40–11.10)	< .001	3.40 (2.55–4.50)*	< .001
Pacemaker	9.16 (60.05–13.35)	< .001	–	–
MCPR	0.64 (0.53–0.78)	< .001	0.50 (0.34–0.72)*	< .001
ECMO	9.28 (7.71–11.11)	< .001	0.55 (0.37–0.80)*	.002

Adjusted for sex, age, witnessed arrest, bystander CPR, place of arrest, cause of arrest, shockable EKG rhythm, prehospital defibrillation, PCI, TTM, Pacemaker, MCPR and ECMO. * Factors included in the final logistic regression model for survival to discharge. OR, odds ratio; CI, confidence interval; CPR, cardiopulmonary resuscitation; PCI, Percutaneous coronary intervention; TTM, Targeted temperature management; MCPR, Mechanical cardiopulmonary resuscitation; ECMO, extracorporeal membrane oxygenation.

Supplementary Table S3. Univariate and multivariate logistic regression analysis of ROSC.

Factor	Univariate OR (95% CI)	*P* value	Adjusted OR (95% CI)	*P* value
Male	0.92 (0.89–0.94)	< .001	0.93 (0.88–0.99)*	.02
Age, years	10.00 (10.00–10.00)	0.49	–	–
Witnessed arrest	2.36 (2.29–2.44)	< .001	20.04 (1.93–2.17)*	< .001
Bystander CPR	0.96 (0.90–10.01)	0.14	0.89 (0.84–0.95)*	< .001
Arrest in public place	0.88 (0.85–0.92)	< .001	–	–
Cardiac origin	0.26 (0.24–0.27)	< .001	0.23 (0.20–0.26)*	< .001
Shockable EKG rhythm	10.07 (10.02–1.11)	.002	1.56 (1.37–1.78)*	< .001
Prehospital defibrillation	0.90 (0.87–0.93)	< .001	0.71 (0.63–0.80)*	< .001
MCPR	0.92 (0.88–0.96)	< .001	0.81 (0.75–0.87)*	< .001

Adjusted for sex, age, witnessed arrest, bystander CPR, place of arrest, cause of arrest, shockable EKG rhythm, prehospital defibrillation, PCI, TTM, Pacemaker, MCPR and ECMO. * Factors included in the final logistic regression model for survival to discharge. OR: odds ratio; CI: confidence interval; CPR: cardiopulmonary resuscitation; MCPR, Mechanical cardiopulmonary resuscitation.

Supplementary Table S4. Comparison between AutoPulse^{TM} and manual CPR

	Manual CPR (N=21458)	AutoPulse^{TM} (N=419)	*P* value
Male	13666 (63.7%)	283 (67.5%)	.12
Age, years	70.2 ± 14.7	70.8 ± 14.0	.45
Witnessed arrest	12325 (59.1%)	257 (62.7%)	.16
Bystander CPR	5537 (77.2%)	138 (70.4%)	.03
Arrest in public place	2892 (17.1%)	56 (16.7%)	.89
Cardiac origin	20247 (94.4%)	395 4.3%)	> .999
Shockable EKG rhythm	2808 (13.2%)	50 (12.0%)	.54
Prehospital defibrillation	4165 (19.4%)	78 (18.7%)	.74
PCI	530 (2.5%)	5 (1.2%)	.13
TTM	761 (3.5%)	16 (3.8%)	.87
Pacemaker	58 (0.3%)	1 (0.2%)	> .999
ECMO	369 (1.7%)	7 (1.7%)	> .999
ROSC	9130 (42.5%)	157 (37.5%)	.04
Survival to hospital discharge	852 (4.0%)	14 (3.3%)	.60
Good neurologic outcome	333 (1.6%)	4 (1.0%)	.43

Values are presented as the means ± standard deviations and frequency (proportion). CPR, cardiopulmonary resuscitation; PCI, percutaneous coronary intervention; TTM, targeted temperature management; ECMO, extracorporeal membrane oxygenation; ROSC, return of spontaneous circulation.

Supplementary Table S5. Comparison between LUCAS^{TM} and manual CPR

	Manual CPR (N=21458)	LUCAS^{TM} (N=5479)	*P* value
Male	13666 (63.7%)	3552 (64.8%)	.12
Age, years	70.2 ± 14.7	70.0 ± 14.8	.36
Witnessed arrest	12325 (59.1%)	3146 (58.9%)	.75
Bystander CPR	5537 (77.2%)	2054 (71.8%)	< .001
Arrest in public place	2892 (17.1%)	742 (16.5%)	.38
Cardiac origin	20247 (94.4%)	5087 (92.8%)	< .001
Shockable EKG rhythm	2808 (13.2%)	665 (12.2%)	.07
Prehospital defibrillation	4165 (19.4%)	1011 (18.5%)	.11
PCI	530 (2.5%)	159 (2.9%)	.08
TTM	761 (3.5%)	276 (5.0%)	< .001
Pacemaker	58 (0.3%)	15 (0.3%)	> .999
ECMO	369 (1.7%)	152 (2.8%)	< .001
ROSC	9130 (42.5%)	2308 (42.1%)	.58
Survival to hospital discharge	852 (4.0%)	194 (3.5%)	.15
Good neurologic outcome	333 (1.6%)	60 (1.1%)	.014

Values are presented as the means ± standard deviations and frequency (proportion). CPR, cardiopulmonary resuscitation; PCI, percutaneous coronary intervention; TTM, targeted temperature management; ECMO, extracorporeal membrane oxygenation; ROSC, return of spontaneous circulation.

Supplementary Table S6. Comparison between Thumper^{TM} and manual CPR

	Manual CPR (N=21458)	Thumper^{TM} (N=130)	*P* value
Male	13666 (63.7%)	86 (66.2%)	.62
Age, years	70.2 ± 14.7	68.1 ± 15.2	.11
Witnessed arrest	12325 (59.1%)	85 (70.2%)	.02
Bystander CPR	5537 (77.2%)	23 (57.5%)	.006
Arrest in public place	2892 (17.1%)	19 (18.1%)	.89
Cardiac origin	20247 (94.4%)	120 (92.3%)	.41
Shockable EKG rhythm	2808 (13.2%)	26 (20.0%)	.03
Prehospital defibrillation	4165 (19.4%)	31 (23.8%)	.25
PCI	530 (2.5%)	4 (3.1%)	.87
TTM	761 (3.5%)	13 (10.0%)	< .001
Pacemaker	58 (0.3%)	3 (2.3%)	< .001
ECMO	369 (1.7%)	3 (2.3%)	.86
ROSC	9130 (42.5%)	58 (44.6%)	.70
Survival to hospital discharge	852 (4.0%)	10 (7.7%)	.053
Good neurologic outcome	333 (1.6%)	6 (4.6%)	.014

Values are presented as the means ± standard deviations and frequency (proportion). CPR, cardiopulmonary resuscitation; PCI, percutaneous coronary intervention; TTM, targeted temperature management; ECMO, extracorporeal membrane oxygenation; ROSC, return of spontaneous circulation.

Supplementary Table S7. Univariate and multivariable logistic regression analyses of survival to hospital discharge during the COVID-19 pandemic.

Factor	Univariate OR (95% CI)	*P* value	Adjusted OR (95% CI)	*P* value
Male	1.46 (1.27–1.67)	< .001	–	–
Age, years	0.97 (0.97–0.97)	< .001	0.98 (0.97–0.99)*	< .001
Witnessed arrest	3.14 (2.69–3.68)	< .001	1.62 (1.22–2.17)*	.001
Bystander CPR	1.26 (0.99–1.61)	.066	–	–
Arrest in public place	1.65 (1.40–1.92)	< .001	1.40 (10.05–1.84)*	.020
Cardiac origin	10.05 (0.81–1.38)	.719	–	–
Shockable EKG rhythm	5.45 (4.79–6.19)	< .001	2.65 (1.97–3.56)*	< .001
Prehospital defibrillation	3.55 (3.13–40.02)	< .001	–	–
PCI	23.87 (20.20–28.19)	< .001	10.24 (7.19–14.56)*	< .001
TTM	17.55 (15.13–20.33)	< .001	8.80 (6.50–11.85)*	< .001
Pacemaker	10.72 (6.44–17.31)	< .001	3.49 (10.07–10.43)*	.030
MCPR	0.91 (0.78–10.05)	.209	–	–
ECMO	7.62 (6.12–9.42)	< .001	–	–

Adjusted for sex, age, witnessed arrest, bystander CPR, place of arrest, cause of arrest, shockable EKG rhythm, prehospital defibrillation, PCI, TTM, Pacemaker, MCPR and ECMO. * Factors included in the final logistic regression model for survival to discharge. OR, odds ratio; CI, confidence interval; CPR, cardiopulmonary resuscitation; PCI, Percutaneous coronary intervention; TTM, Targeted temperature management; MCPR, Mechanical cardiopulmonary resuscitation; ECMO, extracorporeal membrane oxygenation.

Supplementary Table S8. Univariate and multivariate logistic regression analyses of good neurological outcomes during the COVID-19 pandemic.

Factor	Univariate OR (95% CI)	P value	Adjusted OR (95% CI)	P value
Male	2.26 (1.78–2.91)	< .001	–	–
Age, years	0.95 (0.95–0.96)	< .001	0.97 (0.96–0.98)*	< .001
Witnessed arrest	40.01 (30.06–5.34)	< .001	1.42 (0.90–2.30)*	0.14
Bystander CPR	1.90 (1.26–2.97)	0.003	1.98 (1.12–3.72)*	.024
Arrest in public place	20.08 (1.64–2.64)	< .001	1.56 (10.03–2.36)*	.03
Cardiac origin	3.16 (1.68–6.96)	.001	–	–
Shockable EKG rhythm	16.25 (13.10–20.27)	< .001	8.37 (4.98–14.60)*	< .001
Prehospital defibrillation	9.1 (7.38–11.26)	< .001	–	–
PCI	43.43 (350.03–53.82)	< .001	12.49 (80.03–19.51)*	< .001
TTM	10.12 (80.02–12.69)	< .001	2.76 (1.68–4.45)*	< .001
Pacemaker	12.76 (6.51–22.94)	< .001	–	–
MCPR	0.75 (0.57–0.96)	.026	0.54 (0.32–0.87)*	.014
ECMO	10.98 (8.23–14.45)	< .001	–	–

Adjusted for sex, age, witnessed arrest, bystander CPR, place of arrest, cause of arrest, shockable EKG rhythm, prehospital defibrillation, PCI, TTM, Pacemaker, MCPR and ECMO. * Factors included in the final logistic regression model for survival to discharge. OR, odds ratio; CI, confidence interval; CPR, cardiopulmonary resuscitation; PCI, Percutaneous coronary intervention; TTM, Targeted temperature management; MCPR, Mechanical cardiopulmonary resuscitation; ECMO, extracorporeal membrane oxygenation.

Supplementary Table S9. Univariate and multivariable logistic regression analyses of ROSC during COVID-19 pandemic.

Factor	Univariate OR (95% CI)	P value	Adjusted OR (95% CI)	P value
Male	0.93	.002	–	–
Age, years	1.00 (1.00–1.00)	.569	–	–
Witnessed arrest	2.32 (2.21–2.44)	< .001	2.31 (2.18–2.45)*	< .001
Bystander CPR	0.97 (0.88–1.06)	.486	–	–
Arrest in public place	0.89 (0.83–0.95)	.001	–	–
Cardiac origin	0.27 (0.24–0.30)	< .001	0.29 (0.26–0.33)*	< .001
Shockable EKG rhythm	1.07 (1.00–1.15)	.067	1.45 (1.27–1.66)*	< .001
Prehospital defibrillation	0.90 (0.85–0.96)	< .001	0.68 (0.60–0.76)*	< .001
MCPR	0.97 (0.92–1.03)	.336	–	–

Adjusted for sex, age, witnessed arrest, bystander CPR, place of arrest, cause of arrest, shockable EKG rhythm, prehospital defibrillation, PCI, TTM, Pacemaker, MCPR and ECMO. * Factors included in the final logistic regression model for survival to discharge. OR: odds ratio; CI: confidence interval; CPR: cardiopulmonary resuscitation; MCPR, Mechanical cardiopulmonary resuscitation.

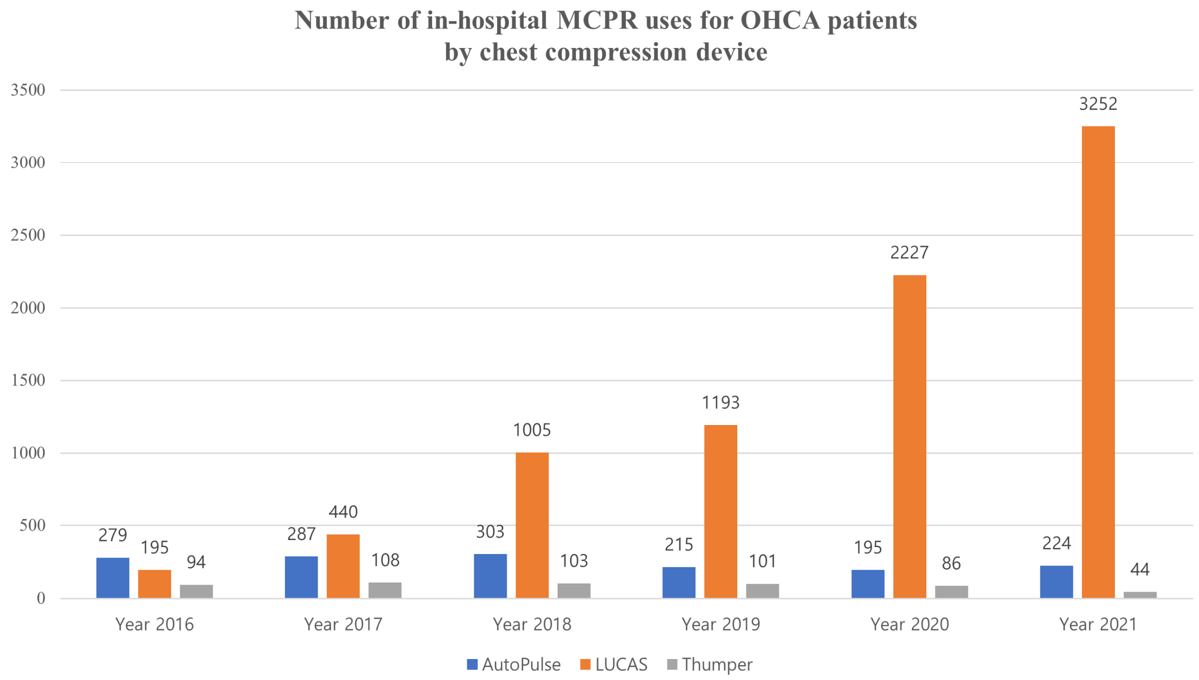

Supplementary Figure S1. Number of in-hospital MCPR uses for OHCA patients by chest compression device
