# Supplementary figures and images for: Assessing the Impact of the Pandemic on Treatment Outcomes for Cardiac Arrest Patients Utilizing Mechanical CPR: A Nationwide Population-Based Observational Study in South Korea
Source: J Pers Med. 2024 Oct 24;14(11):1072. doi: 10.3390/jpm14111072 (PMC11595693; doi:10.3390/jpm14111072)

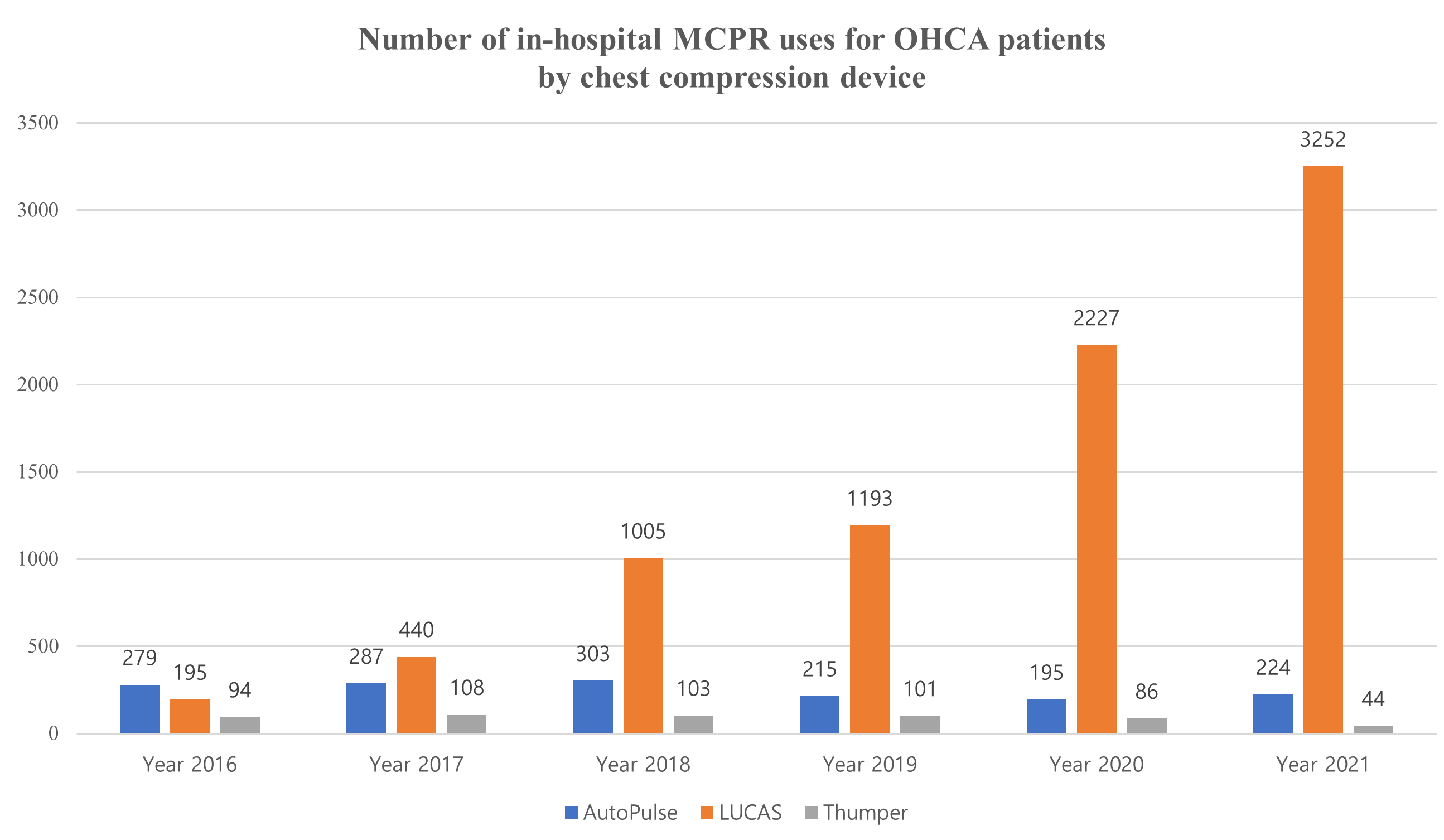

Supplement: Supplementary file 1 [file jpm-14-01072-s001.zip › Supplementary Figure S1.TIF]
